# Biomechanical study of two-level oblique lumbar interbody fusion with different types of lateral instrumentation: a finite element analysis

**DOI:** 10.3389/fmed.2023.1183683

**Published:** 2023-06-23

**Authors:** Yuan Zhong, Yujie Wang, Hong Zhou, Yudong Wang, Ziying Gan, Yimeng Qu, Runjia Hua, Zhaowei Chen, Genglei Chu, Yijie Liu, Weimin Jiang

**Affiliations:** ^1^Department of Orthopaedic Surgery, Dushu Lake Hospital Affiliated to Soochow University, Suzhou, Jiangsu Province, China; ^2^Department of Orthopaedic Surgery, The First Affiliated Hospital of Soochow University, Suzhou, Jiangsu Province, China; ^3^Department of Orthopedics, Union Hospital, Tongji Medical College, Huazhong University of Science and Technology, Wuhan, Hubei Province, China; ^4^Suzhou Medical College, Soochow University, Suzhou, Jiangsu Province, China

**Keywords:** biomechanical study, oblique lumbar interbody fusion, finite element study, endplate stress, Optistruct

## Abstract

**Objective:**

The aim of this study was to verify the biomechanical properties of a newly designed angulated lateral plate (mini-LP) suited for two-level oblique lumbar interbody fusion (OLIF). The mini-LP is placed through the lateral ante-psoas surgical corridor, which reduces the operative time and complications associated with prolonged anesthesia and placement in the prone position.

**Methods:**

A three-dimensional nonlinear finite element (FE) model of an intact L1–L5 lumbar spine was constructed and validated. The intact model was modified to generate a two-level OLIF surgery model augmented with three types of lateral fixation (stand-alone, SA; lateral rod screw, LRS; miniature lateral plate, mini-LP); the operative segments were L2–L3 and L3–L4. By applying a 500 *N* follower load and 7.5 *Nm* directional moment (flexion-extension, lateral bending, and axial rotation), all models were used to simulate human spine movement. Then, we extracted the range of motion (ROM), peak contact force of the bony endplate (PCFBE), peak equivalent stress of the cage (PESC), peak equivalent stress of fixation (PESF), and stress contour plots.

**Results:**

When compared with the intact model, the SA model achieved the least reduction in ROM to surgical segments in all motions. The ROM of the mini-LP model was slightly smaller than that of the LRS model. There were no significant differences in surgical segments (L1–L2, L4–L5) between all surgical models and the intact model. The PCFBE and PESC of the LRS and the mini-LP fixation models were lower than those of the SA model. However, the differences in PCFBE or PESC between the LRS- and mini-LP-based models were not significant. The fixation stress of the LRS- and mini-LP-based models was significantly lower than the yield strength under all loading conditions. In addition, the variances in the PESF in the LRS- and mini-LP-based models were not obvious.

**Conclusion:**

Our biomechanical FE analysis indicated that LRS or mini-LP fixation can both provide adequate biomechanical stability for two-level OLIF through a single incision. The newly designed mini-LP model seemed to be superior in installation convenience, and equally good outcomes were achieved with both LRS and mini-LP for two-level OLIF.

## 1. Introduction

Oblique lumbar interbody fusion (OLIF) is becoming increasingly prevalent in treating lumbar spinal degenerative diseases due to its minimal invasiveness, indirect decompression, high fusion rate, and ability to allow fast rehabilitation (1, 2). Compared to traditional transforaminal lumbar interbody fusion (TLIF), the OLIF procedure has many advantages. First, the large cage used in OLIF surgery provides a larger area, reducing contact pressure between the cage and end plate and potentially preventing cage subsidence; second, the large contact area between the bone graft and end plate may increase the fusion rate (3–5). In addition, the large cages also produce satisfactory outcomes for the correction of spinal force lines: the lumbar lordosis and disk height are substantially restored. Then, the foramen is indirectly enlarged with nerve root release (6).

According to the current mainstream understanding of the procedure, OLIF incorporates supplementary fixation to increase postoperative stability (7–10). The types of supplementary instruments in OLIF surgery typically fall into one of two categories: insertion of the cage without supplementary fixation (stand-alone, SA) technique and percutaneous bilateral pedicle screw fixation after cage implantation. However, the commonly used internal fixations have limitations that should be addressed. Some studies have found that the SA technique is associated with inadequate initial stability inducing cage subsidence, a longer bed-rest period, and a high reoperation rate (11). Although percutaneous bilateral pedicle fixation is considered the gold standard due to its prominent biomechanical performance, surgeons still need to reposition the patient in the prone posture under anesthesia, which can prolong the operation, require an additional incision, and increase costs (9). Minimally invasive pedicle screw placement also exposes surgeons to higher doses of radiation than traditional hand-free pedicle screw placement or other types of instrumentation (12).

Upon deepen understanding of lumbar degenerate mechanism and avoiding the occurrence of adjacent segment disease (ASD), an increasing number of lumbar degenerative disease (LDD) require two level fusion using OLIF technique during clinical practice (13–15). Several researchers have reported that the one level OLIF procedure could also yield promising prognosis, in which they augmented the surgical segment laterally with rod screw or angulated locking plate without additional incision (10, 16). However, the study on biomechanical stability of plates or rod-screw fixation in two level OLIF could rarely be seen; and it is not clear that which kind of lateral fixation suits best in the two-level OLIF surgery. Given the greater understanding of the lumbar degenerative mechanism and avoidance of the occurrence of ASD in recent years, an increasing number of LDDs have required two-level fusion using the OLIF technique in clinical practice (13–15). Several researchers have reported that the one-level OLIF procedure can also yield a promising prognosis, in which the surgical segment is augmented laterally with a rod screw or angulated locking plate without additional incisions (10, 16). However, studies on the biomechanical stability of plate or rod-screw fixation in two-level OLIF are rare, and it is not clear which kind of lateral fixation is best suited for two-level OLIF surgery.

To resolve the issues above, researchers have mainly adopted two methods to study spine biomechanics: *in vitro* specimen tests and finite element analysis (FEA). Investigating *in vitro* specimens is a fundamental research method in biomechanics (17, 18). However, experiments are time consuming due to difficulties in acquiring and preprocessing the specimen. Moreover, the paraspinal muscles and ligaments are prone to decay, and the test apparatuses vary across studies, leading to a lack of repeatability. With the commercialization of finite element (FE) software such as Abaqus or ANSYS, many scholars have begun to use the finite element method to investigate biomechanics (19–22). Different spine surgical FE models have been established to simulate the transient postoperative state by instantiating various boundaries and loading conditions, from which data such as Von Mises stress of different components are extracted to evaluate stability. The finite element method is not only superior in controlling the experimental parameters but also repeatable. However, to date, FE software has been unable to accurately mimic the paraspinal soft tissues, and validated FE calculation results that represent the trends in the data are of exact values. Cai et al. studied intradisk pressure by constructing a threedimensional lumbar model of L1-L3 and found that abnormal load and motion may accelerate the degeneration of the adjacent segment (22). Liu et al. created a finite element model of the lumbar spine from L2-L5 that simulated lateral lumbar fusion surgery. They found that stand-alone fixation may generate higher endplate stress than other supplementary fixations, which may increase the risk of cage subsidence (20). Given advancements in computer science and mathematics, the precision of FEA is increasing, and its value has become increasingly accepted by a large number of academics.

In our practice, we commonly choose the lateral-rod screw (LRS) as a supplementary instrument due to its effectiveness and inexpensiveness, but it has two obvious shortcomings. First, the high raised tail of the pedicle screw and rod may injure the psoas major postoperatively. Second, to obtain an optimal holding force with the screw and avoid damaging the vertebral segmental blood vessels and the nerve plexus, the screw needs to be installed carefully. Due to the lack of specific installation tools and the need to make deep surgical incisions, it can be frustrating and time consuming for surgeons to complete this step. Compared to single-level OLIF, two-level scenarios may be even more difficult to perform (e.g., due to the use of a long rod within a small skin incision). To overcome these limitations, we have developed a thin angulated locking plate appropriate for OLIF surgery that combines the concept of a traumatic locking plate with the principle of easy installation. In this study, we mainly compared the biomechanical properties of two-level OLIF surgery augmented with this miniature lateral plate (mini-LP) with the LRS using the FE method.

## 2. Methods

### 2.1. Construction of full L1–L5 lumbar model

**Figure 1:**
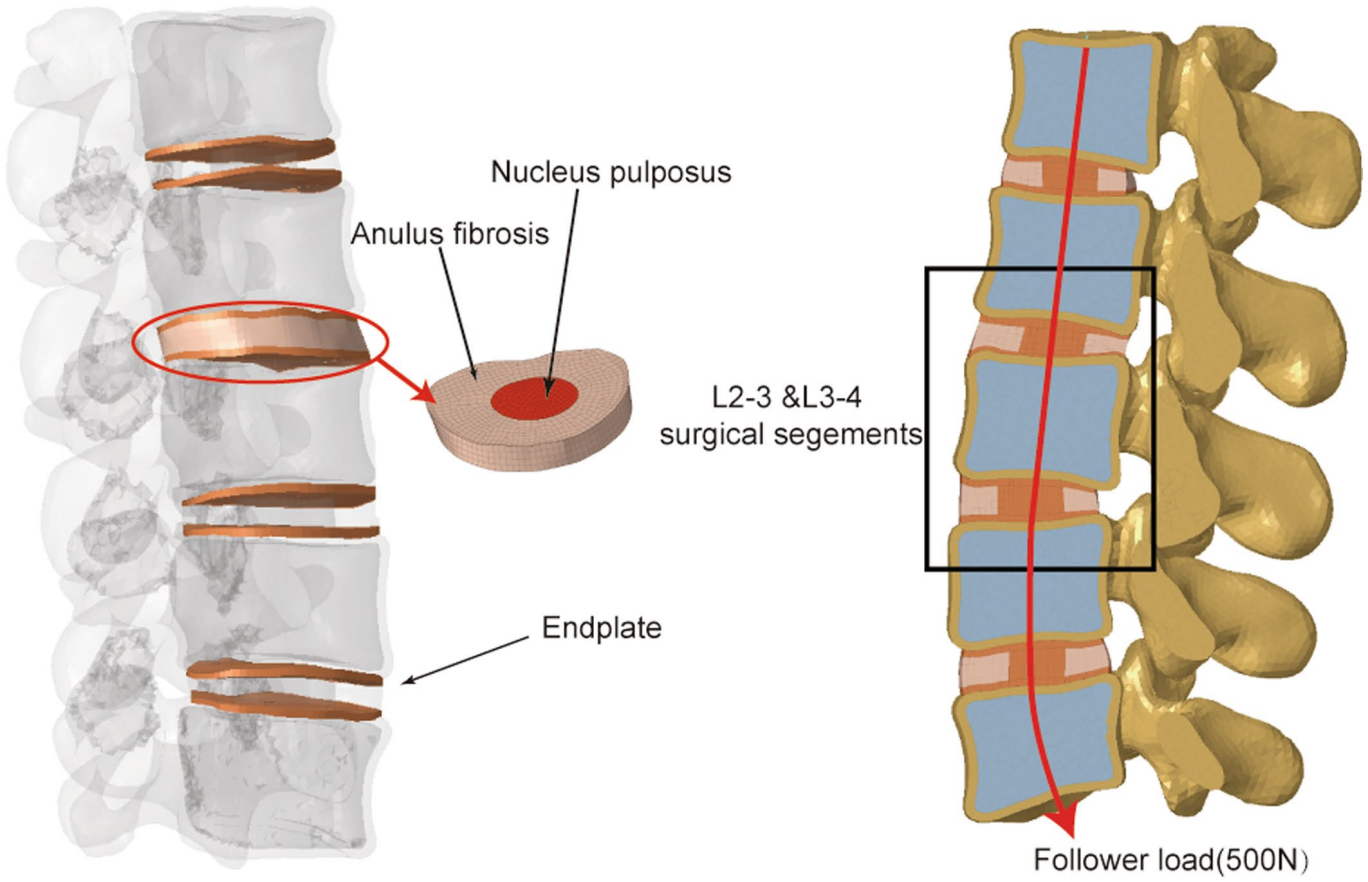
Illustration of L1–L5 intact finite element model.

The computed tomography (CT) data of a 40-year-old man were collected from our hospital’s Department of Radiology in Digital Imaging and Communications in Medicine (DICOM) format and served as the basis for the models employed in the study. The included patient had no history of lumbar infectious illnesses, malignancies, degenerative diseases, or abnormalities. This study was approved by the institutional review board of Dushu Lake Hospital affiliated to Soochow University (No. DF-2021-042). The patient who agreed to participate in the research signed an informed authorization form, and any information identifying the individuals has been anonymized. To create three-dimensional models, the L1–L5 CT DICOM-format images were loaded into Mimics Research 19.0 (Materialise NV, Leuven, Belgium) software. In this software, we used the “segmentation” function to isolate the lumbar spine from the entirety of the images to generate the original lumbar STL files. Then, we imported the acquired STL files into Geomagic Studio 2013 (3D Systems, Inc., Rock Hill, South Carolina, USA) to polish the model. During the process, the tools “Construct Patches” and “Grid and Fit Surfaces” were implemented, and the modified models were then exported in STEP format. The STEP files were then imported into SolidWorks 2017 (Dassault Systmes SolidWorks Corporation, Waltham, Massachusetts, USA). The involved elements were created, including cortical bone, cancellous bone, posterior structures, endplate, annulus fibrosus, nucleus pulposus, articular cartilage and ligaments (Figure 1). Finally, the FE meshes of the different spinal components were constructed using HyperWorks 2022 (Altair Engineering Corp, Michigan, USA) computer-aided engineering (CAE) software. Finally, FE analysis software Optistruct (Altair Engineering Corp, Michigan, USA) was utilized to biomechanically simulate the lumbar spine model. Tetrahedral (vertebral body, facet) and hexahedral (intervertebral disk, endplate) elements were used to mesh all parts of the finite element model except for the ligaments. After performing a mesh sensitivity test, an average mesh size of 1.5 mm was chosen. A total of 268,349 elements and 70,282 nodes composed the complete model.

The intervertebral disc, ligament system, and L1–L5 vertebral bodies were all incorporated into the finite element model. The vertebral bodies each included the cortical bone, cancellous bone, bony structures of the posterior column, and end plates. The cortical bone was 2-mm thick, and the end plates were 1-mm thick (19, 22). The nucleus pulposus composed 44% of the intervertebral disc, while the annulus fibrosus composed 56% (23). The vertebral body, facet joints and posterior elements were defined as isotropic, homogeneous elastic materials (24). Mooney-Rivlin and Yeoh hyperelastic materials were used to model the nucleus pulposus and annulus fibrosus, respectively (25, 26). The paraspinous ligaments included the anterior longitudinal ligament (ALL), posterior longitudinal ligament (PLL), ligamentum flavum (LF), interspinous ligament (ISL), supraspinous ligament (SSL), capsular ligament (CL), and intertransverse ligament (ITL), which were configured as linear-spring elements that were only subjected to tensile loading (27, 28). The material properties of the models are listed in Supplementary Table S1.

### 2.2. Development of OLIF surgical models

**Figure 2:**
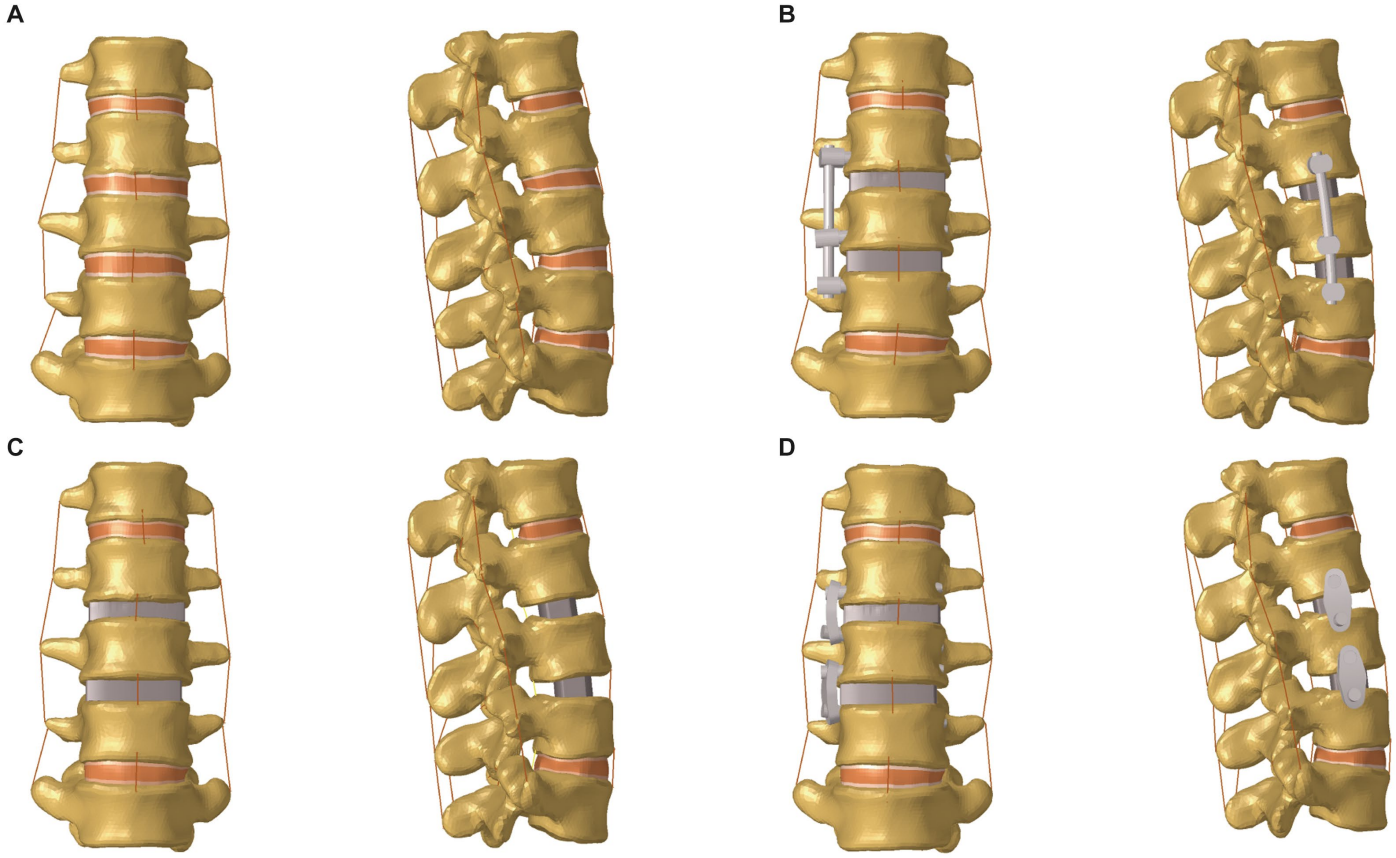
Front and lateral views of the lumbar spine FE model (L1–L5). **(A)** Intact model; **(B)** OLIF with lateral rod-screw fixation (OLIF + LRS) model; **(C)** Stand-alone OLIF (SA OLIF) model; **(D)** OLIF with mini-lateral plate fixation (OLIF + mini-LP) model; OLIF, oblique lumbar interbody fusion.

**Figure 3:**
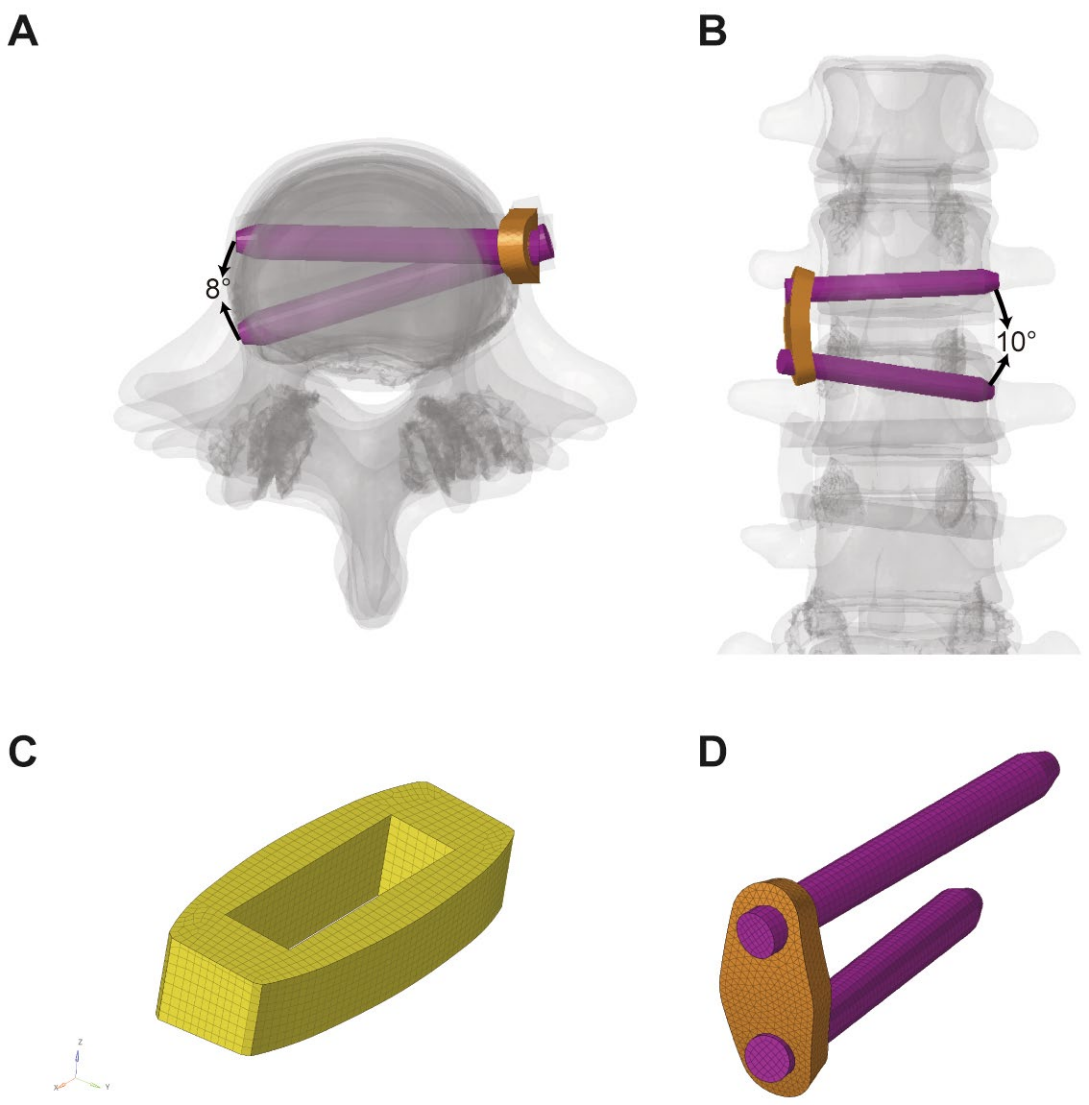
Illustration of mini-LP system. **(A)** Cross section view; **(B)** Coronal view; **(C)** Cage; **(D)** mini-LP; mini-LP, mini-lateral plate.

The involved intervertebral disc nucleus, annulus fibrosus, and nearby endplates were all removed to mimic the OLIF surgery. Two separate cages were inserted into the L2–3 and L3–4 intervertebral spaces and different types of supplementary constructs were installed separately. We defined the cage’s property as Polyetheretherketone (PEEK), and the property of rod, screw and angulated locking plate as titanium (Figure 2): (I) SA: two cages (45 × 20 × 9 mm^3^, 8°) were implanted without the use of any additional fixations; (II) LRS: following the placement of two cages (45 × 20 × 9 mm^3^, 8°), three pedicle screws (length: 45 mm, outer diameter: 6.5 mm) were bicortically fixed onto three vertebrae with a rod connection (length: 70 mm, diameter: 5 mm); (III) mini-LP: after the placement of two cages (45 × 20 × 9 mm3, 8°), two oval-shaped lateral plates (30 × 13 × 5 mm^3^) coupled with four locking screws (length: 45 mm, outer diameter: 6.5 mm) were implanted on the three vertebrae. The mini-LP consists of an oval-shaped self-locking plate and two angled (sagittal and coronal) locking screws (Figure 3). The mini-LP is especially designed to be installed between the L2–L5 intervertebral spaces and is compatible with the OLIF cage. The distances between the nearby lumbar segmental arteries determined the length of the plates needed to protect the segmental arteries (29), and the width was set to fit the surgical corridor (30). Many OLIF clinical anatomic studies have provided evidence for us to design the plate: we set the lateral plate width at 13 mm, the length at 30 mm (in 2-mm increments), and the thickness at 5 mm. The mini-LP has a streamlined curved shape, with a 1 radian arc on the coronal plane to fit the anatomical surface of the lateral vertebrae. The screws were designed as fully threaded cortical screws with a 6.5 mm diameter that ranged in length from 40 mm to 55 mm (in 2-mm increments). The thread of the screw body is a fishbone spur-type tapered thread that effectively increases the screw-bone interface and the screw holding power. The primary idea behind the design of the mini-LP is the trajectory of the multidirectional locking screws. To achieve the greatest biomechanical structural stabilization and increase the contact area of the screw-bone interface, we specifically set two lateral screws at an angle of 10 to the horizontal centerline of the plate and an angle of 5–8 to the vertical centerline of the plate.

### 2.3. Contact, boundary, and loading conditions

Due to muscle strength and trunk weight, the human lumbar spine can support large compressive stresses *in vivo*. These compressive pressures play a crucial role in strengthening the lumbar spine’s load-bearing capacity to preserve its mechanical stability. Patwardhan et al. created the “follower load” concept; in their experiment, a compressive preload was administered to a multisegmental lumbar spine specimen without inducing its collapse (31). Based on their experiment, up to a 1,000-*N* follower load was applied on the specimen, which had reached the maximum limit of the lumbar spine. Generally, the follower load of the normal human spine is up to 500 *N* (31). In our FE model, we introduced a 500 *N* compressive load along the center of each vertebra to mimic normal paraspinal muscles and body weight. The follower load was composed of physiological compression along the lumbar spine axis (Figure 1).

In our study, we implied the follower load described in previous studies (17, 22). Specifically, coupling points were set at the center of the L1-L5 upper endplates and used to create connector elements. Then, a 500 *N* follower load was applied on each vertebra through the connector elements.

The nonlinear-static biomechanical analysis was implemented by Optistruct 2022. The boundary and loading conditions were established using HyperWorks 2022 based on previous research (19, 22, 23, 32, 33). The contact type between the intervertebral disk and endplate, endplate and vertebrae, rod and pedicle screw, plate and locking screw, and cage and bony endplate was set to binding mode “Tie.” The contact type between facet joints was set to frictionless mode “Slide.” The linear spring-like “CBUSH1D” element was used to simulate all seven types of ligaments, namely, the anterior longitudinal ligament, posterior longitudinal ligament, ligamentum flavum, intertransverse ligament, interspinous ligament, supraspinous ligament, and joint capsule structures. Each element has two points connected to where they should exist according to the lumbar anatomy. The elastic parameters were set by referring to the previous literature, and the stiffness was set to 8.7, 5.8, 15.4, 0.2, 10.9, 2.4, and 15.8 *N/mm*, respectively (21, 23). While maintaining the follower load (500 *N*), a moment load of 7.5 *Nm* was applied to the superior surface of the L1 upper endplate to mimic six directional movements [flexion (FLEX), extension (EX), right bending (RB), left bending (LB), right axial rotation (RAR), and left axial rotation (LAR)]. During the loading process, the bottom of the L5 vertebrae was fully constrained in six degrees of freedom.

### 2.4. Data collection

The computations were performed in finite element modelling (FEM) software Optistruct 2022 after the models were correctly constructed in HyperWorks 2022 using the exact boundary and loading conditions mentioned above. The range of motion (ROM), which refers to the rotational angle of each adjacent lumbar spine segment under the six physiological motions, was recorded. Furthermore, the maximum equivalent von Mises stress of the plates, rods, screws, and cages, the peak contact force of the bony endplate (PCFBE), the peak equivalent stress of fixation (PESF), and the peak equivalent stress of the cage (PESC) were measured to evaluate the potential for structural failure and cage subsidence in the different OLIF surgical models. For data analysis, the calculations were repeated for the mesh convergence test to reduce the possibility of mesh size-related mistakes. In this study, the results that demonstrated a stable solution with a variation of less than 5% when the mesh size was altered three times were recognized as acceptable values and documented. The incorrect stress concentration was ignored in this study. Optistruct is a sophisticated FE program that has been utilized in a wide range of engineering and medical simulations ranging from simple linear analyses to complicated nonlinear problems in FE studies (34, 35).

## 3. Results

### 3.1. Finite element model validation

**Figure 4:**
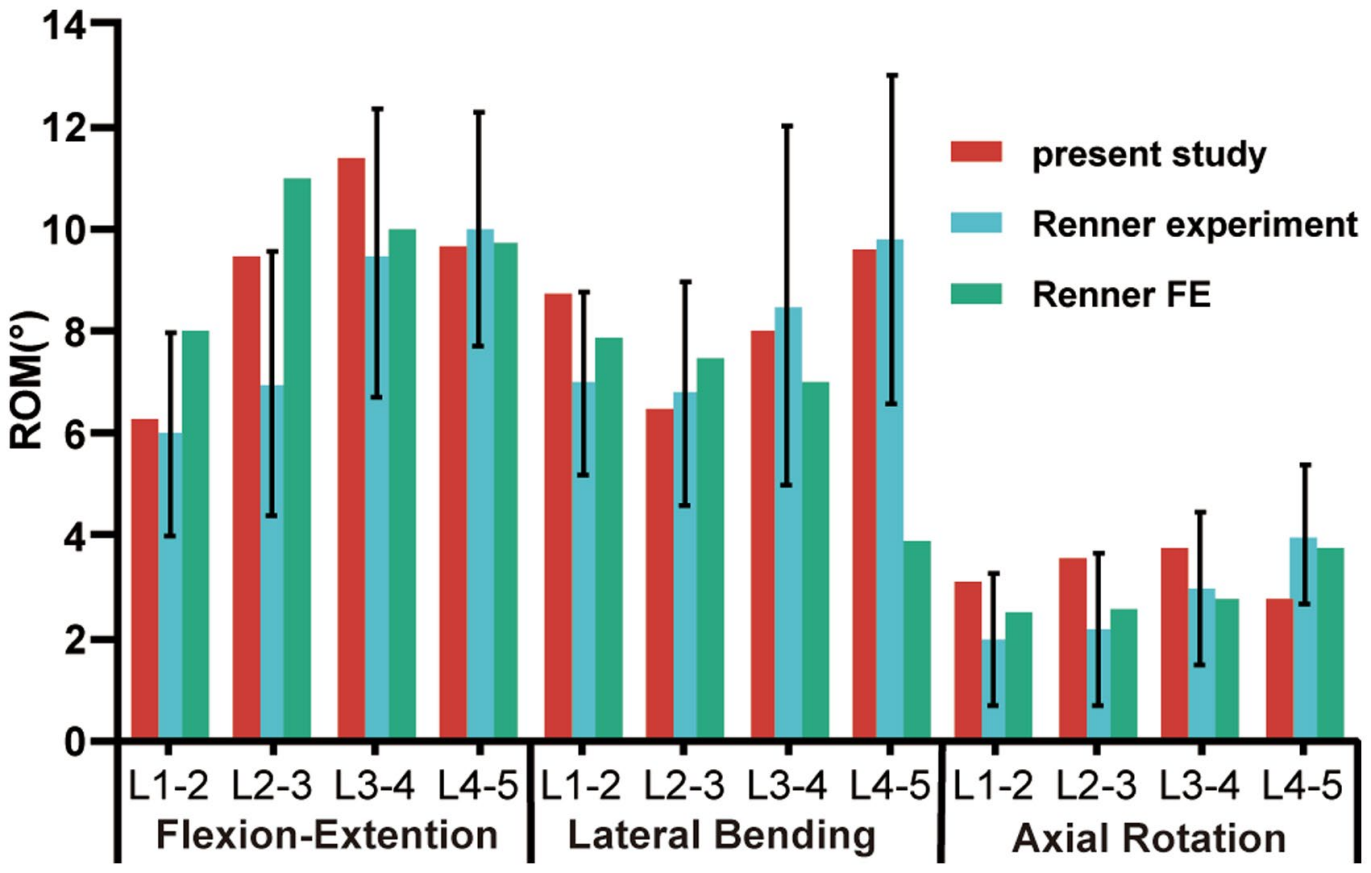
Comparison of *in vitro* and FE ROM results for each motion unit in the intact lumbar model with those of Renner’s study during flexion-extension, lateral bending, and axial rotation; ROM, range of motion.

The L1–L5 segmental ROMs for the six direction motions of the intact model were measured under a 500 *N* axial follower compression preload and a 7.5 *Nm* moment load and compared them to the experimental and FE analyses implemented by Renner et al. (36) As shown in Figure 4, the ROMs of the present model were within one standard deviation of those in Renner’s study (36). Consequently, our FE model of the intact L1-L5 model was considered validated, allowing its further use in the biomechanical analysis of the lumbar spine under varying conditions.

### 3.2. Range of motion

**Figure 5:**
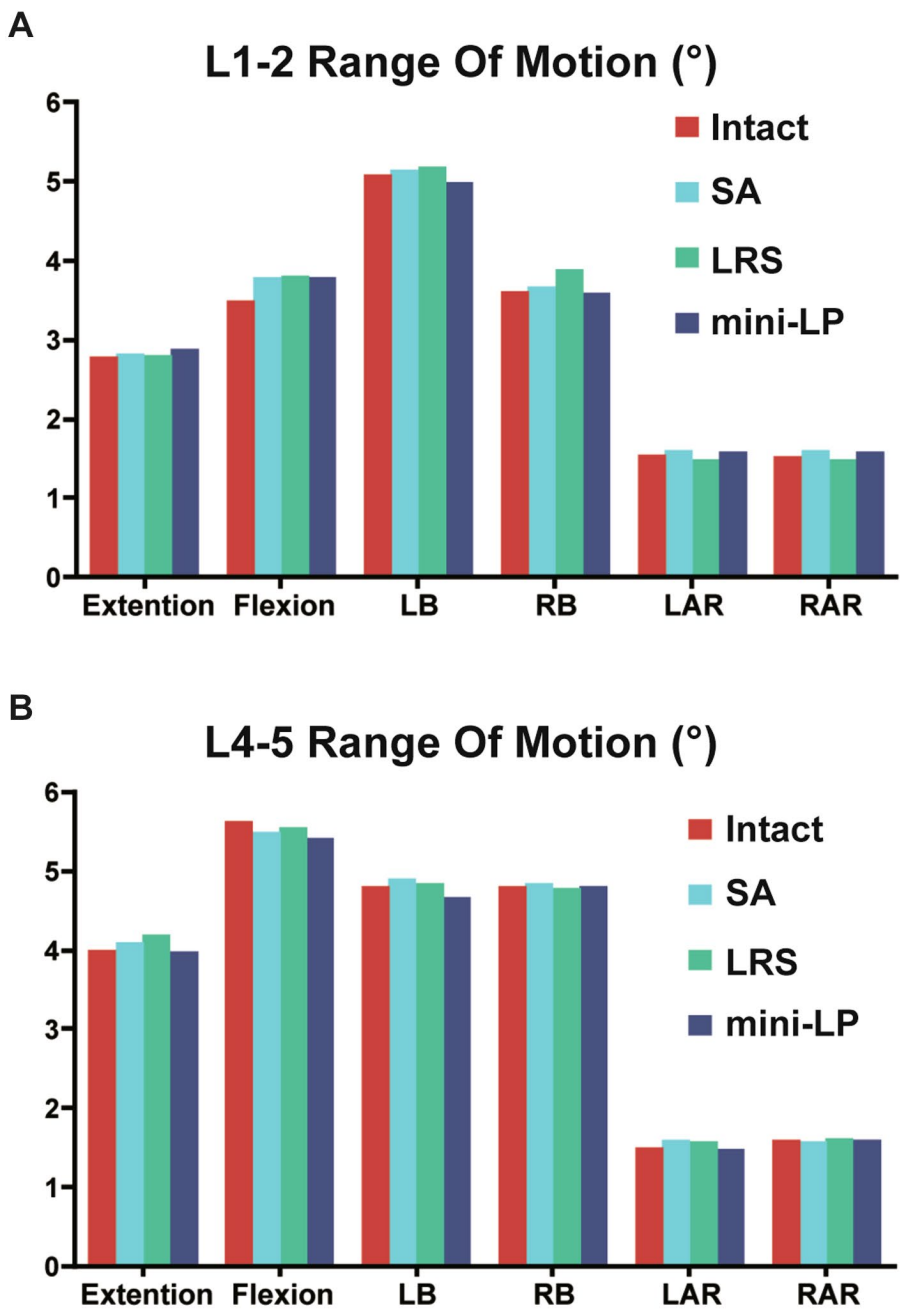
Range of motion at the parasurgical segments (L1–L2, L4–L5) of the four models under six directional loading conditions. **(A)** L1–L2 segment; **(B)** L4–L5 segment; LB, left bending; RB, right bending; LAR, left axial rotation; RAR, right axial rotation; SA, stand-alone; LRS, lateral rod-screw; mini-LP, miniature lateral plate.

**Figure 6:**
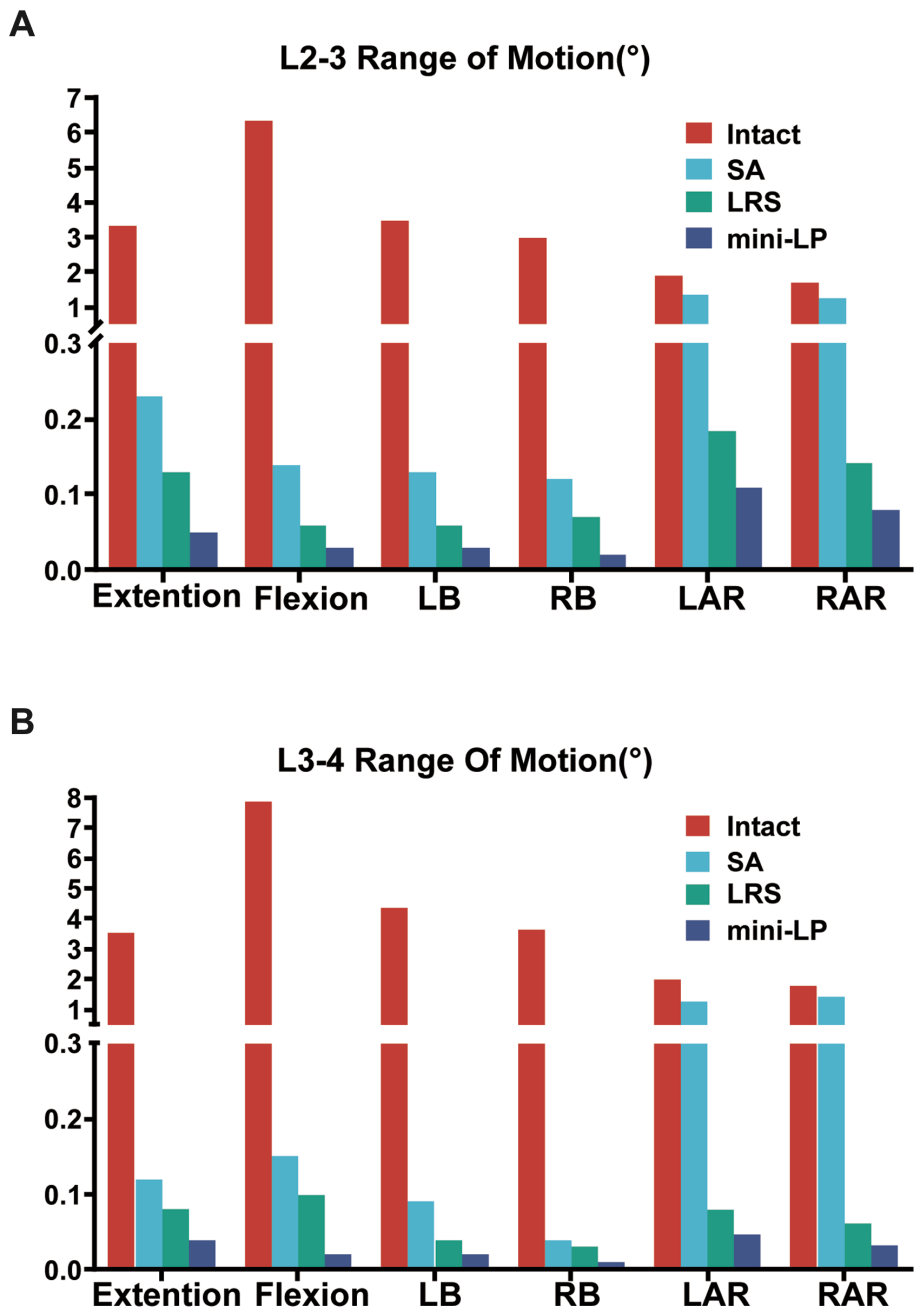
Range of motion at the surgical segments (L2–L3, L3–L4) of the four models under six directional loading conditions. **(A)** L2–L3 segment; **(B)** L3–L4 segment; LB, left bending; RB, right bending; LAR, left axial rotation; RAR, right axial rotation; SA, stand-alone; LRS, lateral rod-screw; mini-LP, miniature lateral plat.

The ROMs of segments L1–L5 under six loading conditions, including the intact and three OLIF surgical models, are shown in Figures 5, 6. Comparing the intact model to the surgical models, there were no obvious differences in the ROM of the parasurgical segments (Figure 5). The ROMs of parasurgical segment L1–L2 in the LRS model showed a slight upward trend in the lateral bending posture and a slight decrease in the axial rotation posture relative to the other surgical models (Figure 5A). The ROMs of the parasurgical segment L4–5 in the mini-LP model showed a slightly decreasing trend in the flexion-extension posture relative to the other surgical models (Figure 5B). The ROMs of the surgical segments (L2–L3, L3–L4) were reduced in all postures for all OLIF surgical models relative to the intact model, as shown in Figure 6. In the stand-alone OLIF model, the cage provided the smallest restriction to the ROMs of both surgical segments in all six postures. The L2–L3 and L3–L4 ROMs were 93.13 and 96.59% in extension, 97.80 and 98.09% in flexion, 96.29 and 97.94% in LB, 96 and 98.90% in RB, 28.95 and 37.50% in LAR, and 26.47 and 20.56% in RAR, respectively, of the intact model. The ROM of the surgical segments in each posture was larger in the SA model than in the other two OLIF surgery models. Additionally, the ROM of the surgical segments was smaller in the mini-LP model than in the LRS model, but, the variances between those two were not significant (Figure 6).

### 3.3. Peak contact force of bony endplate

**Figure 7:**
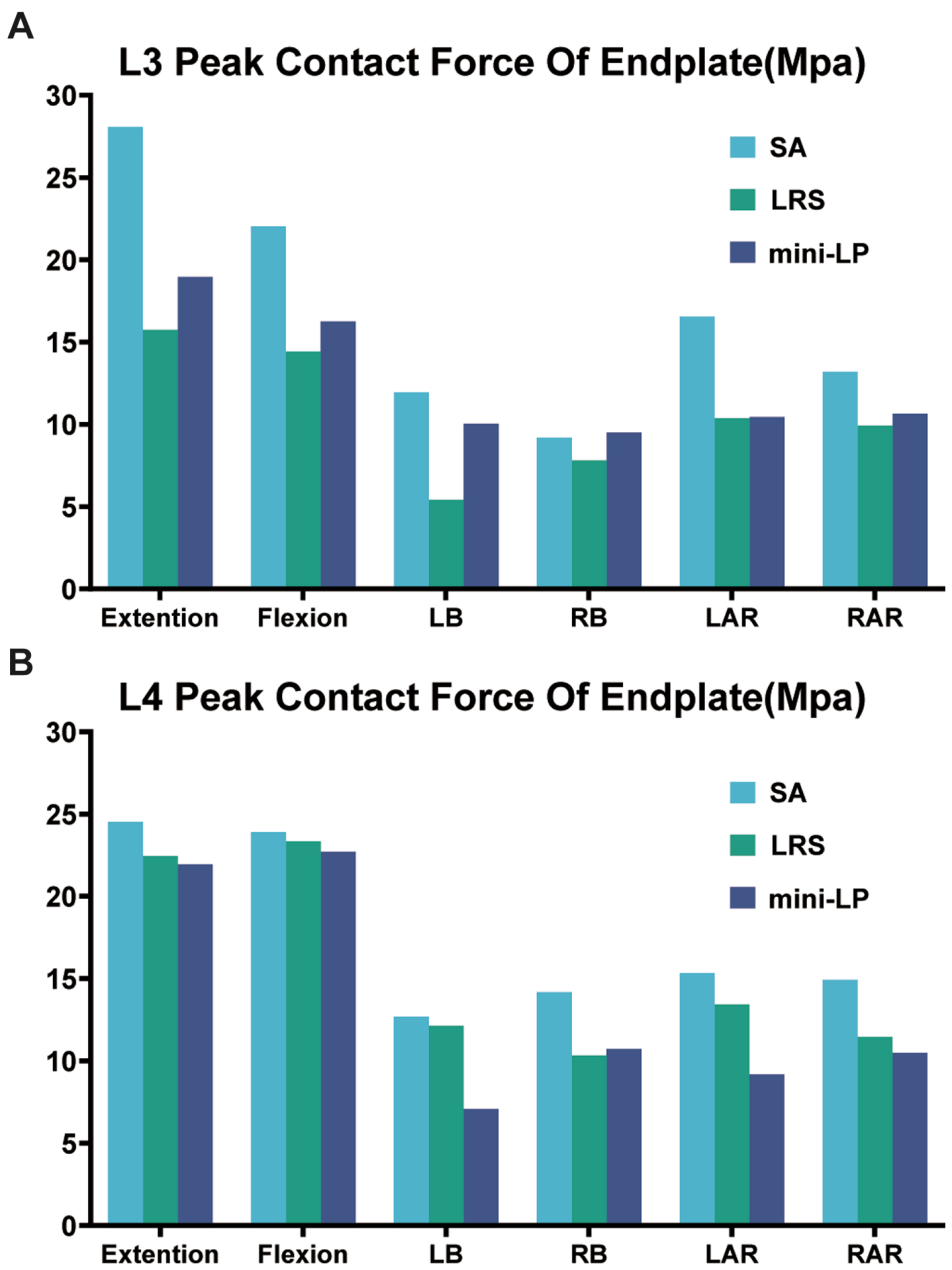
Peak contact force of the bony endplate (PCFBE) of the three OLIF surgical models. **(A)** L3 superior endplate; **(B)** L4 superior endplate; SA, stand-alone; LRS, lateral rod-screw; mini-LP, miniature lateral plate.

**Figure 8:**
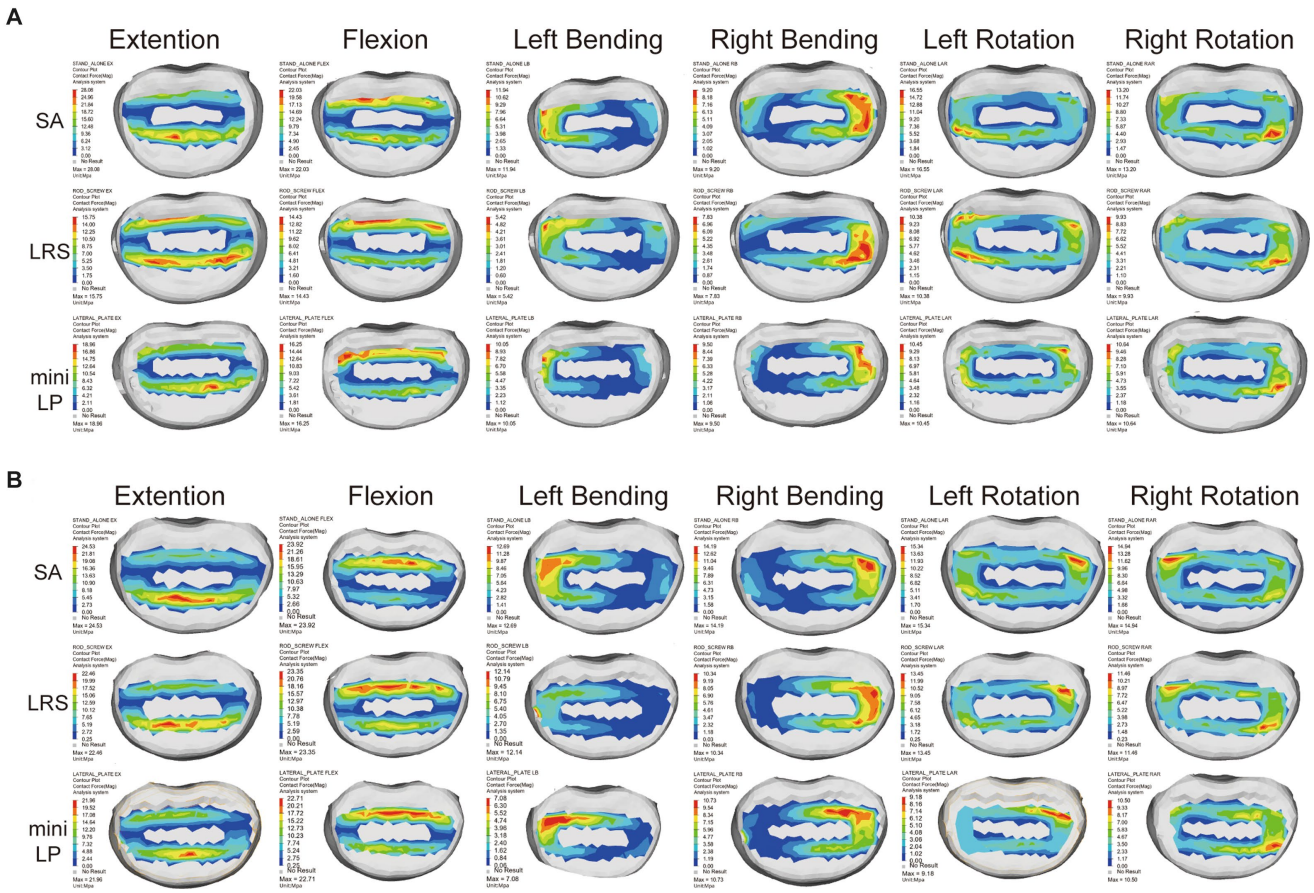
Contact force distribution for the L3 **(A)** and L4 **(B)** superior end plates in the three OLIF surgical models under various loading conditions. SA, stand-alone; LRS, lateral rod-screw; mini-LP, miniature lateral plate.

The PCFBE values for the L3 and L4 superior end plates of the three OLIF surgical models under various loading conditions are displayed in Figures 7, 8 shows the contact force distribution of the L3 and L4 superior end plates, which clearly demonstrates that the maximum contact stress of the superior end plate was concentrated at the rim of the cages where the bony end plate just meets the cage. The PCFBE values of the L3 superior end plate in the LRS model were 15.75, 14.43, 5.42, 7.83, 10.38, and 9.93 Mpa under extension, flexion, LB, RB, LAR, and RAR loading conditions, respectively; these values were 43.91, 34.50, 54.61, 14.89, 37.28, and 24.77% lower than those of the SA model, respectively. The PCFBE of the L3 superior end plate in the mini-LP model was 18.96, 16.25, 10.05, 10.45, and 10.64 Mpa under extension, flexion, LB, LAR, and RAR loading conditions, respectively, which were 32.48, 26.24, 15.83, 36.86, and 19.39% lower than those of the SA model, respectively. However, the PCFBE under the RB loading condition in the mini-LP model was 9.5 Mpa, which was 0.03% higher than that of the SA model. Overall, the PCFBEs of the L3 superior end plate in the LRS model were lower than those of the mini-LP model (Figure 7A). The PCFBE values of the L4 superior end plate in the LRS model were 22.46, 23.35, 12.14, 10.34, 13.45, and 11.46 Mpa under extension, flexion, LB, RB, LAR, and RAR loading conditions, respectively, which were 8.44, 2.38, 4.33, 27.13, 12.32, and 23.29% lower than those of the SA model, respectively. The PCFBE of the L4 superior end plate in the mini-LP group was 21.96 Mpa in extension, 22.71 Mpa in flexion, 7.08 Mpa in LB, 10.73 Mpa in RB, 9.18 Mpa in LAR and 10.50 Mpa in RAR loading conditions, which were 10.48, 5.06, 44.21, 24.38, 40.16, and 29.72% lower than those in the SA model, respectively. Overall, the PCFBEs of the L4 superior end plate in the mini-LP model showed a descending trend relative to those of the LRS model under all loading conditions except RB (Figure 7B).

### 3.4. Peak equivalent stress of cage

**Figure 9:**
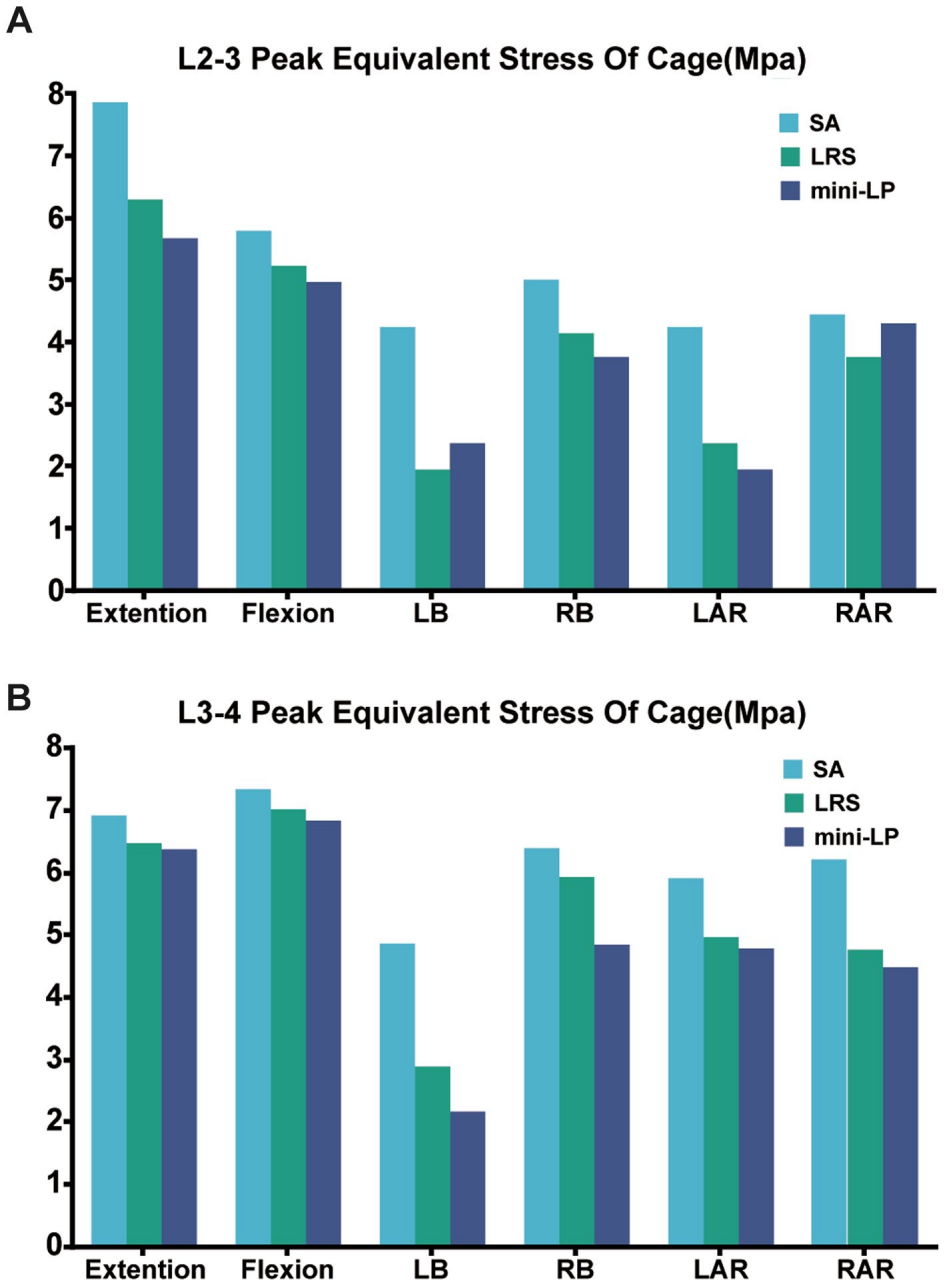
Peak equivalent stress of the cage (PESC) in the three OLIF surgical models. **(A)** L2–L3 cage; **(B)** L3–L4 cage; SA, stand-alone; LRS, lateral rod-screw; mini-LP, miniature lateral plate.

**Figure 10:**
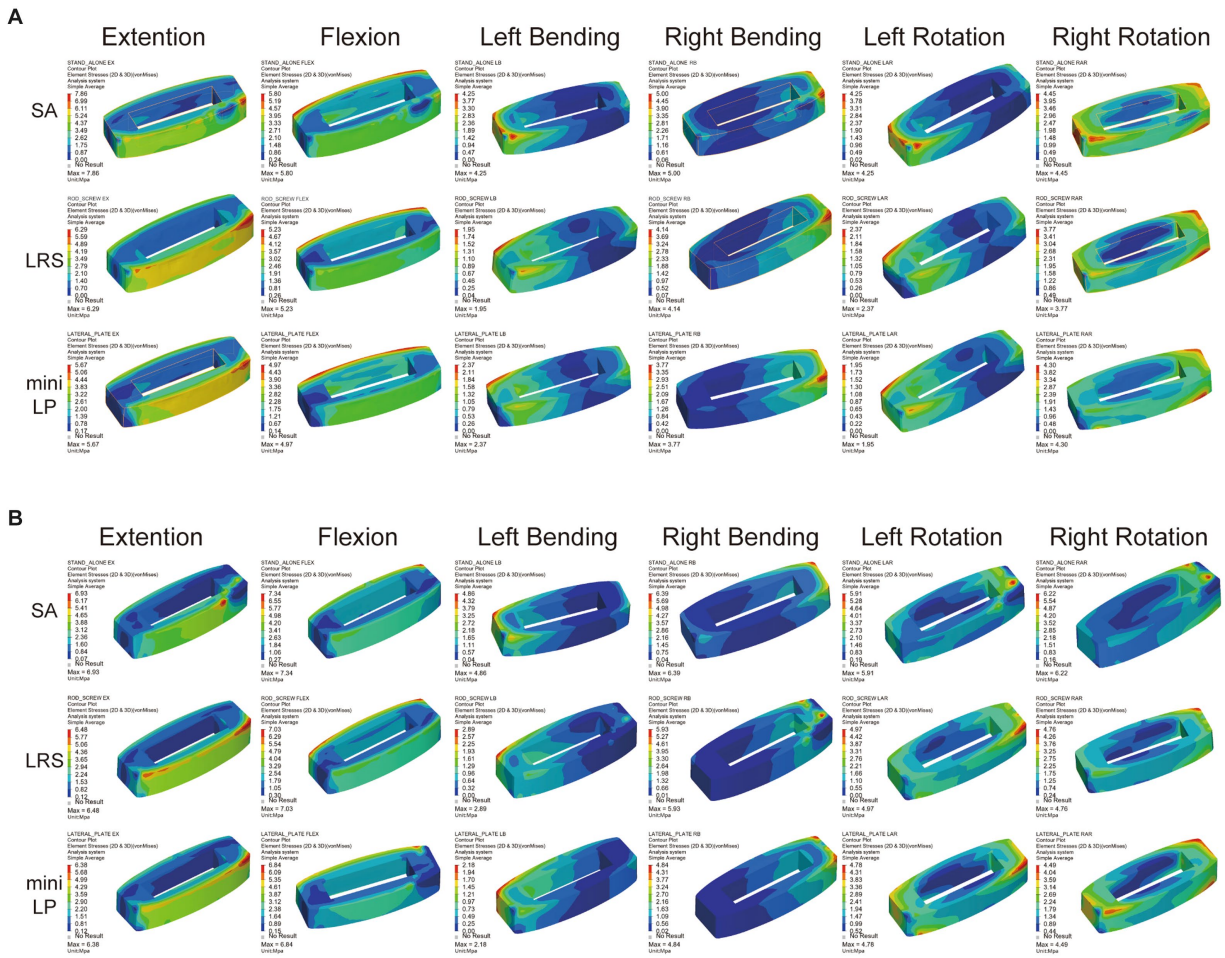
Equivalent stress distribution for the cages in the three OLIF surgical models (SA, LRS, and mini-LP) under six loading conditions. **(A)** L2–L3 cage; **(B)** L3–L4 cage.

The PESC values for the L2–L3 and L3–L4 cages of the three OLIF surgical models under the six loading conditions are displayed in Figure 9. The SA model exhibited the greatest cage stress, especially in the extension and flexion postures, which decreased following the insertion of the internal fixation device. The L2–L3 cage stress of the LRS model was slightly higher than that of the mini-LP model under all loading conditions except the LB and RAR conditions (Figure 9A). In addition, compared with the LRS model, the mini-LP model had 1.54, 2.70, 24.57, 18.38, 3.82, and 5.67% lower L3-L4 cage stress during extension, flexion, LB, RB, LAR, and RAR, respectively (Figure 9B). Figure 10 shows the nephogram of the two cages’ equivalent stress under various loadings. The highest von Mises stress was mainly distributed at the periphery of the cage, consistent with the results of the PCFBE distribution.

### 3.5. Peak equivalent stress of fixiation

**Figure 11:**
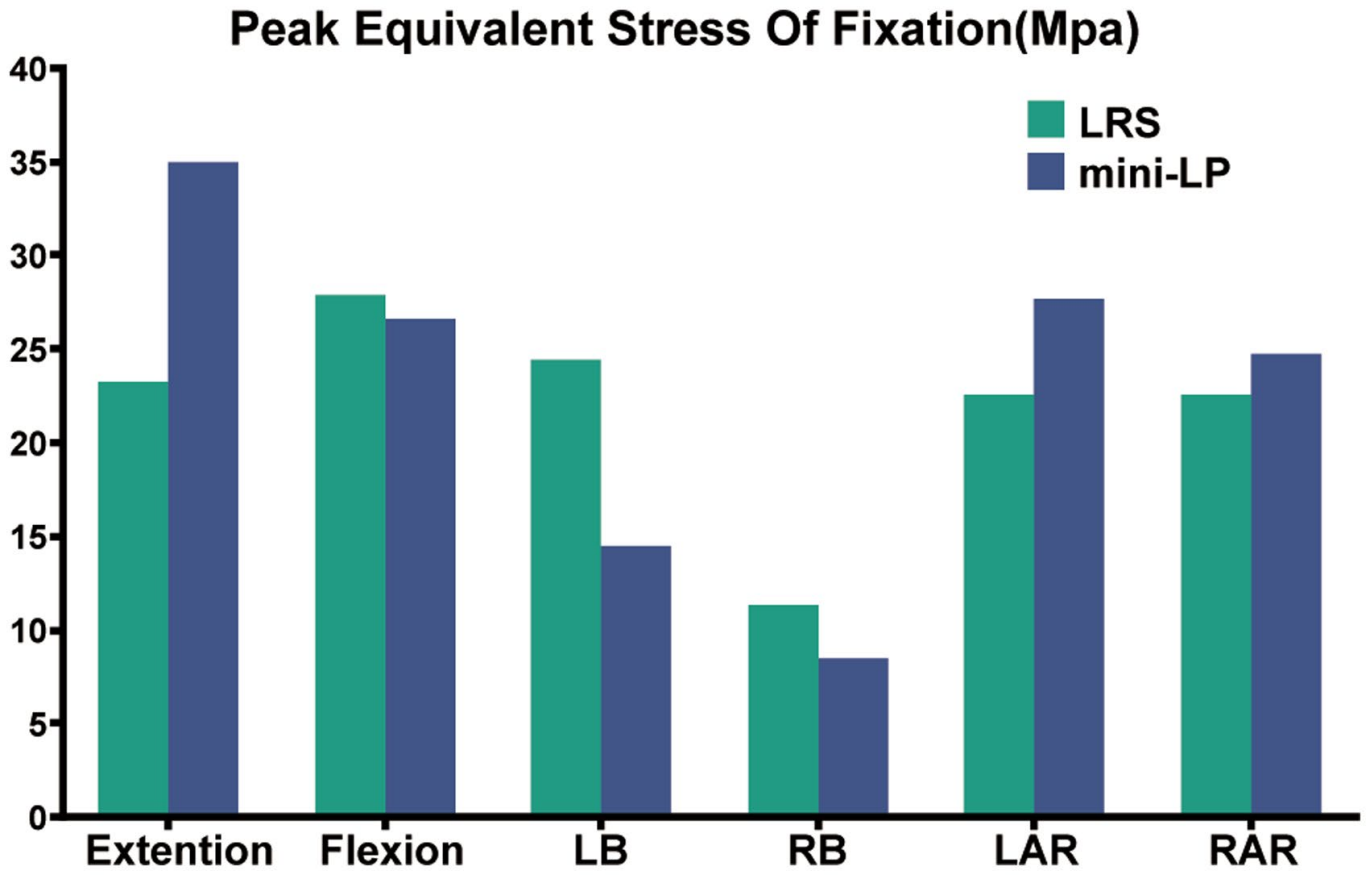
Peak equivalent stress of fixation (PESF) in two OLIF surgical models (LRS, mini-LP) under six loading conditions.

**Figure 12:**
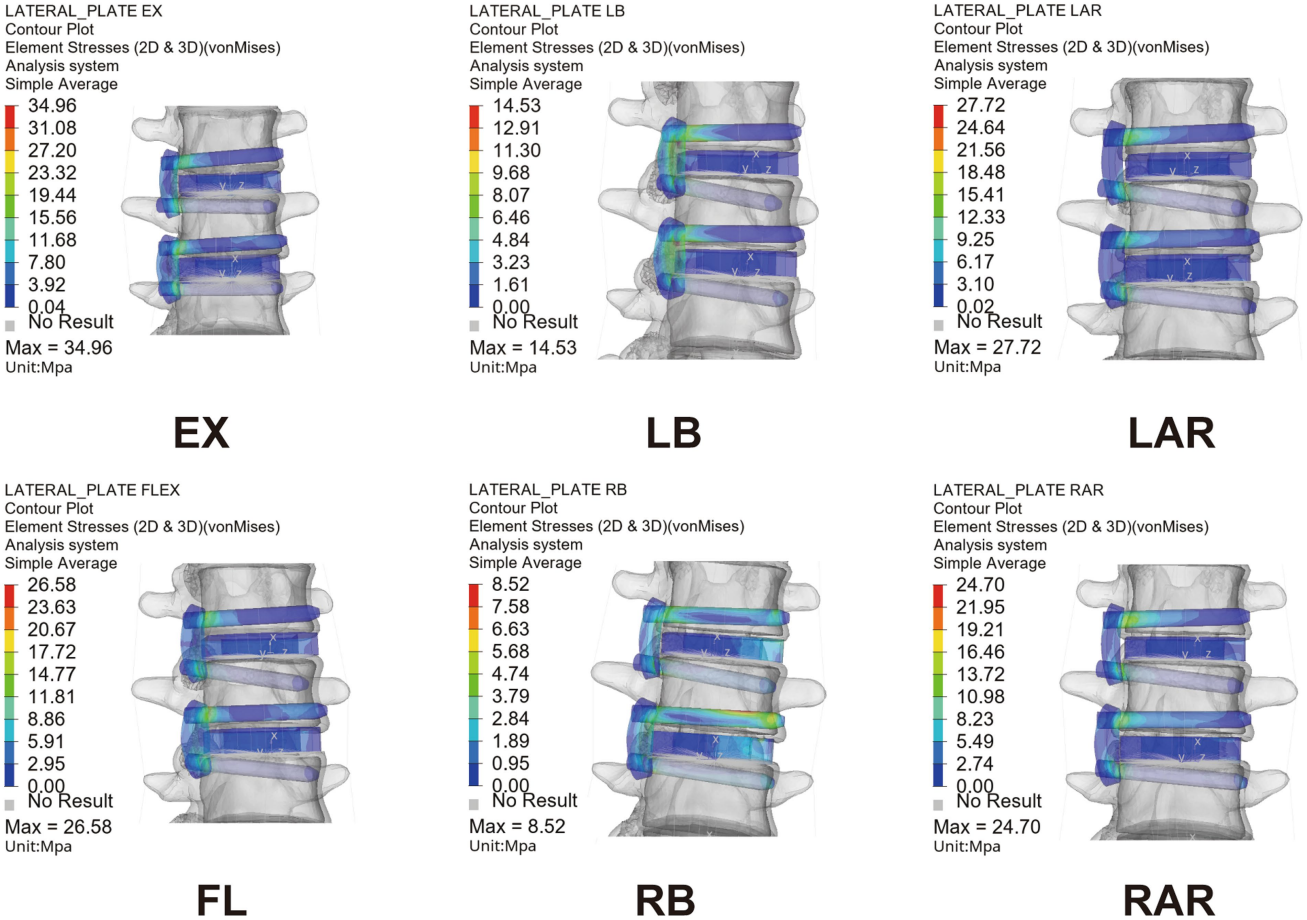
Equivalent stress nephogram of the mini-LP OLIF surgical model under six loading conditions. EX, extension; FL, flexion; LB, left bending; RB, right bending; LAR, left axial rotation; RAR, right axial rotation.

Figure 11 shows the PESF values of lateral fixation, suggesting that the PESF of the mini-LP model was lower than that of the LRS model in regard to flexion, LB and RB loading. However, the opposite was observed when the model was subjected to extension, LAR and RAR loading. Both lateral fixation models endured much lower stress than the yield and fatigue stress of titanium-based internal fixation reported in the related literature (37). Figure 12 shows the fixation stress distribution of the mini-LP model. We can see that the maximum stress was mainly distributed at the contact areas where the screw and the plate intersected.

## 4. Discussion

A key part of this research was to determine how our novel mini-LP fixation functions biomechanically. There are two major approaches to study biomechanical properties: cadaveric experiments and finite element analysis. Biomechanical experiments on cadavers are difficult to perform because there are specific limits regarding how the cadaver should be prepared as well as what lab equipment should be utilized. With the development of a series of finite element software programs, the transformation from lumbar spine CT scans to finite element models has become simple and convenient, paving the way for biomechanical analysis to prevail. Simulations of various fixation models, comparisons of how surgical segments perform and stress analyses of each structure under physiologic loading can assist us in determining how internal fixation functions.

In recent decades, OLIF surgery has become increasingly popular among spine surgeons, as a safe surgical corridor was discovered and established with proficiency. In contrast to traditional lateral lumbar interbody fusion (LLIF), surgeons can safely reach the target intervertebral space through the corridor between the major neurovascular plexus and the psoas, which decreases the risk of intrapsoas plexus injury and allows preservation of the paraspinal tissue to a certain degree (38), resulting in a less invasive and more economical procedure (39). We usually chose simple lateral instrumentation as internal fixation in the OLIF procedure. This fixation eliminates the need to injure the paraspinal muscles while meeting the needs for instant postoperative lumbar spinal stability. In our study, the biomechanical features for two types of commonly used lateral instrumentation systems associated with double surgical segments of the OLIF procedure were investigated for the first time. During two-level OLIF surgery, the installment of lateral fixation can be time consuming and difficult; therefore, we designed a miniature lateral plating system for convenient installment during the procedure while accounting for biomechanical stability.

In our study, four important biomechanical parameters (ROM, PCFBE, PESC, and PESF) were measured to assess the stability and feasibility of the instrumentation we designed for OLIF surgery. It is essential to address postoperative cage subsidence and displacement, as these can determine the success or failure of OLIF surgery. Instability of the surgical segment in lumbar fusion surgery can result in nonunion and pseudoarthrosis, producing irreversible chronic back pain and severely affecting patient recovery (38, 40, 41).

Regardless of the procedure, the first rule of lumbar fusion is to establish a mechanically stable environment for the fusion segment by reducing the ROM of the target segment (30, 42, 43). In the current investigation, all surgical models, regardless of the use of supplementary fixation, increased the surgical segment’s stability relative to that of the intact spine model, and the ROM of the two surgical segments in all surgical models was dramatically smaller than that of the intact model. A smaller surgical segment ROM indicates better segmental stability in OLIF surgery to a certain extent. We discovered that the SA model was associated with larger ROMs, suggesting that the surgical segments with cages alone and no fixation were less stable than those of the two supplementary instrument-based models, which may impair the possibility of interbody fusion. The LRS and mini-LP devices both effectively reduced the ROMs of the surgical segments, but the screw trajectory of the mini-LP can vary in both the sagittal and coronal planes and could provide two-column (anterior, middle) fixation better than the middle column-only fixation of the LRS system. This could be the reason why the mini-LP showed superiority in ROM reduction, especially in extension-flexion and axial rotation loading conditions. Huang et al. constructed several single-level OLIF FE models, including a novel anatomical lateral plate, and found that the lateral plate fixation system provided better stability than that acquired from other models (lateral rod-screw and lateral rod-screw plus facet screw) (30). Wang et al. also found that their newly designed oblique lateral locking plate system (OLLPS) achieved better ROM reduction than the lateral rod-screw models (42). Our results are in agreement with those in the literature, which indicates that lateral plating fixation for OLIF procedures could yield superior biomechanical results than lateral rod-screw instrumentation.

Compared to those of the intact model, the ROM changes of the segments adjacent to the L2–L3 and L3–L4 surgical segments were not obvious in the OLIF models. From a biomechanical standpoint, fusion surgery immobilizes a functioning joint, which significantly increases the segment’s stiffness. Theoretically, the ROMS of the parasurgical segments would increase to compensate for the loss of ROM in the surgical segment. However, that was not the case in our study; perhaps our FE simulation only depicted the immediate postoperative stability, and changes in the ROMs of the parasurgical segments require a gradual process including ligaments, facet joints or muscles.

The PCFBE of each OLIF model with supplementary instrumentation was lower than the PCFBE of the SA OLIF model (Figure 7). The abnormal peak contact stress may result in endplate deterioration or even risk accelerating whole-spine degeneration over time, which could eventually affect the supporting force toward the cage, inducing cage subsidence (20). Macki et al. identified a 10.2% incidence of subsidence in lateral lumbar interbody fusion (44). The stress-growth curve of vertebral body cells indicates that a higher compressive stress is associated with a lower possibility of fusion (45). Steffen et al. found that the yield strength of the bony end plate was correlated with several factors, including age, bone mineral density and the normalized endplate coverage area (46). Our test results showed that the highest PCFBE was 27 Mpa in the SA model under extension loading, which is lower than the yield strength of the bony end plate reported in Steffen’s test. The patients’ bone quality and multilevel fusion are both dangerous factors toward cage subsidence (20, 47, 48). Therefore, by installing the fixation device after the cage was inserted, the construct shared the stress of the cage, eventually reducing the PCFBE among osteoporotic patients specifically. We ignored the thread types of the screw by simply define the contact type of bone-screw as ‘Tie’ to achieve convergence during calculating process according to the previous studies (31, 36, 37). Karakasli et al. (49) developed a biomechanical test to measure the pullout strength of different types of pedicle screws. They found that fully threaded cortical screws had the strongest grasp strength compared to other thread designs. Liu et al. (50) tested the pullout strength of three different thread typed pedicle screws, they found that a combination of the conical and dual-core/dual-thread designs may achieve optimal postoperative screw stability in healthy vertebrae. Jendoubi et al. (51) have designed a FE test, they have found that single-thread screws exhibit better pullout strength than double-thread screws. In this study, the thread of the screw body is a fishbone spur-type tapered thread that effectively increases the screw-bone interface and the screw holding power. Our findings are consistent with previous FE studies (22, 30, 42). Both the mini-LP and LRS provided satisfactory stability for surgical segments by reducing the PCFBE, particularly under lateral bending and axial rotation loads (Figure 7).

The PESC was larger in the stand-alone OLIF model than in the OLIF models with supplementary fixation (Figure 9). The larger PESC may cause injury to the neighboring endplates, resulting in an unnatural rise in end plate stress and possibly degeneration. Destruction of the endplate biomechanical environment increases the risk of cage subsidence and intervertebral space collapse (20, 48). Cage subsidence is a common complication after any kind of lumbar fusion surgery, and the reduction in disc height is often accompanied by adjacent segment degeneration (ASD) (22, 52–54). Once the cage subsides and the disc height diminishes, the endplate and cancellous bone in the surgical segment may endure additional injury, especially the inferior endplate, which may result in alterations to the biomechanics of the adjacent segments (33). Moreover, previous *in vitro* and FE experiments have indicated that the PESC values reflect, to a certain extent, the capacity to resist cage subsidence and preserve disc height (30, 43, 55). We used PEEK as the cages in our FE model due to the favorable mechanical properties, low density (i.e., radiolucency), and excellent chemical resistance. Another popular material for interbody cages is titanium and its associated alloys. McGilvray et al. (56) designed a 3D-printed porous titanium alloy (PTA) cage, and the bone growth through porous structures in PTA group was observed, which was better than that of the PEEK group. Campbell et al. (57) also found that 3D-printed PTA cages had a significantly lower subsidence rate, which may due to their higher or earlier fusion rate compared to PEEK cages in ASD surgery. Additionally, bone density, cage shape, age, and applied distraction may result in increased subsidence rates as well. In our investigation, the bisegmental PESC values of the LRS model and mini-LP model were comparable and substantially lower than those of the SA model under lateral bending and axial rotation loading overall. Thus, the capacity of these two models to resist cage subsidence and maintain disc height was relatively similar.

The PESF values in the LRS model and mini-LP model have distinct characteristics. The mini-LP seemed to have an advantage under lateral bending and flexion loadings in terms of lower PESFs, while the LRS prevailed during extension and axial rotation loading (Figure 11). Song et al. conducted an FE study on OLIF surgery and found that the PESF of the lateral plate was larger than that of other fixation methods, with a maximum lateral plate stress ranging from 33.16 to 191.4 Mpa under different loading conditions (58). These values are larger than those determined in our research (8.52–34.94 Mpa), presumably because only the L3–L4 segment was tested in their investigation. In contrast, we established an L1–L5 whole lumbar model in which we simulated OLIF within two consecutive segments (L2–L3, L3–L4), allowing the stress to be distributed more evenly on two lateral plates and the vertebrae. Selection of either posterior or lateral fixation as a supplementary procedure in OLIF surgery has long been disputed. Theoretically, many FE and *in vitro* studies have shown that the traditional gold standard bipedicle fixation and other kinds of posterior fixation systems indeed provide more stability for the lumbar spine after OLIF surgery than lateral fixation systems (30, 42, 58, 59). However, practically, posterior supplementary instrumentation (1) involves additional use of the prone posture, extra incisions, more blood loss and surgery time and (2) is detrimental to the integrity of the posterior column. Liu et al. discovered that OLIF with lateral rod fixation with a deliberately designed crew insertion angle showed good postoperative outcomes (8). Li et al. showed that the lateral fixation technique in OLIF can achieve 1-stage intervertebral fusion and minimize the operative time (10). Compared to posterior fixation, both LRS and mini-LP instrumentation can offer appropriate stability in OLIF surgery using a single incision and achieve a satisfactory postoperative outcome.

There are several limitations in our study. First, our FE model did not imitate the screw loosening and paraspinal muscles, which might have resulted in the inaccurate depiction of the real biomechanical changes of the lumbar spine and the stress distribution of different spinal components. Second, the ligaments were modeled as one-dimensional linear spring elements due to the complexity of their actual structures and the difficulty in recreating three-dimensional structures, which may have reduced the accuracy of the simulation results. Third, variability is present in the geometric morphology of each individual’s lumbar spine, including disc height, disc degeneration, and facet joint degeneration. Our intact model was established based on the CT scan of a single person with some simplifications during modeling. Therefore, to a certain extent, our FE model can only represent the biomechanical changes of the lumbar spine in response to varied loads. Finally, OLIF surgery is usually conducted on degenerated spines. We constructed the intact spine based on a healthy person without considering spinal degeneration and the associated decline in bone mineral density. Consequently, the results may not be applicable to people with significant bone deterioration. Further studies will evaluate the effects of mechanical simulation under overload conditions, deformities, and diseased conditions on spinal stability.

## 5. Conclusion

The current study constructed an intact L1–L5 lumbar spine based on parameters in previous literature. In addition, we designed a mini-lateral plate for two-level OLIF surgery. Moreover, we established three surgical models (SA, LRS, and mini-LP) based on an intact L1–L5 FE lumbar spine. The present study compared the biomechanical parameters (ROM, PCBE, and PESC) of three types of bisegmental OLIFs model under different loading conditions. We found that OLIF with supplementary fixation involving either an LRS or mini-LP system could achieve better stability than SA OLIF. By analyzing the ROM, PCBE, and PESC PESF data of the surgical segments in the lateral fixation models, we concluded that both the mini-LP and LRS systems could effectively provide necessary stability for the surgical segments and prevent cage subsidence under various loading conditions. Considering the small size and convenience of installation, the mini-LP could be considered as a supplementary fixation device for implantation via a single incision and position to improve bisegmental OLIF surgery.

## Data availability statement

The original contributions presented in the study are included in the article/Supplementary material, further inquiries can be directed to the corresponding authors.

## Ethics statement

The studies involving human participants were reviewed and approved by the institutional review board of Dushu Lake Hospital affiliated to Soochow University (No. DF-2021-042), and all methods were performed in accordance with the Declaration of Helsinki. The patients/participants provided their written informed consent to participate in this study.

## Author contributions

YZ, GC, YL, and WJ designed the study. YZ, GC, and YL performed all experimental procedures. YZ, GC, YW, and HZ carried out the data analysis. GC, YW, ZG, and ZW involved in the data processing and checking. YZ, GC, and WJ wrote the manuscript. GC, YZ, WJ, YQ, and RH contributed to the revise of manuscript. All authors have approved the final version of the article.

## Funding

The study was supported by the Natural Science Foundation of Jiangsu Province (BK20200199), National Natural Science Foundation of China (32101103), China Postdoctoral Science Foundation (2021M702412), and Suzhou Medical Innovation and Application Program (SKY2022126).

## Conflict of interest

The authors declare that the research was conducted in the absence of any commercial or financial relationships that could be construed as a potential conflict of interest.

## Publisher’s note

All claims expressed in this article are solely those of the authors and do not necessarily represent those of their affiliated organizations, or those of the publisher, the editors and the reviewers. Any product that may be evaluated in this article, or claim that may be made by its manufacturer, is not guaranteed or endorsed by the publisher.
