# Supplementary material for: Biomechanical study of two-level oblique lumbar interbody fusion with different types of lateral instrumentation: a finite element analysis
Source: Front Med (Lausanne). 2023 Jun 23;10:1183683. doi: 10.3389/fmed.2023.1183683 (PMC10345158; doi:10.3389/fmed.2023.1183683)
Supplement: Supplementary file 1 [file Table_1.docx]

**Table S1** Material properties of the involved components.

| Component | Young’s modulus E (Mpa) | Poisson’s ratio |
| --- | --- | --- |
| **Bone** |  |  |
| Cortical bone | 12000 | 0.3 |
| Cancellous bone | 100 | 0.3 |
| Cartilage endplate | 1000 | 0.3 |
| Cortical endplate | 12000 | 0.3 |
| Facet joint | 500 | 0.3 |
| Intervertebral disc nucleus pulposus | Mooney–Rivlin, C1=0.12, C2=0.03, D=0.3 |  |
| Annulus fibrosus | Yeoh, C10=0.0146, C20=-0.0189, C30=0.041, D=0.3 |  |
| **Ligament system**  Anterior longitudinal ligament (ALL) | 20 | 0.3 |
| Posterior longitudinal ligament (PLL) | 20 | 0.3 |
| Ligamentum flavum (LF) | 19.5 | 0.3 |
| InteLRSpinous ligament (ISL) | 12 | 0.3 |
| Supraspinous ligament (SSL) | 15 | 0.3 |
| IntertransveLRSe ligament (ITL) | 59 | 0.3 |
| Capsular ligament (CL) | 32.9 | 0.3 |
| **Implant**  Cage (polyetheretherketone) | 3500 | 0.3 |
| Pedicle screw and rods (Ti-6A1-4V) | 110000 | 0.3 |
| Lateral plate and screws (Ti-6A1-4V) | 110000 | 0.3 |
